# Resveratrol Supplementation in Schizophrenia Patients: A Randomized Clinical Trial Evaluating Serum Glucose and Cardiovascular Risk Factors

**DOI:** 10.3390/nu8020073

**Published:** 2016-01-29

**Authors:** Karine Zortea, Viviane C. Franco, Lenise P. Francesconi, Keila M. M. Cereser, Maria Inês R. Lobato, Paulo S. Belmonte-de-Abreu

**Affiliations:** 1 Schizophrenia Program, Hospital de Clínicas de Porto Alegre, Porto Alegre 90035-903, RS, Brazil; vcarvalhofranco@gmail.com (V.C.F.); le.petter@ig.com.br (L.P.F.); keila.cereser@uol.com.br (K.M.M.C.); mirlobato@gmail.com (M.I.R.L.); pbabreu@gmail.com (P.S.B.A.); 2 Postgraduate Program in Medicine: Psychiatry, Universidade Federal do Rio Grande do Sul, Porto Alegre 90035-903, RS, Brazil

**Keywords:** cardiovascular disease, cholesterol, resveratrol, schizophrenia

## Abstract

Background: Patients with schizophrenia (SZ) are generally overweight or obese and have several metabolic disorders. Additionally, such patients have a lower life expectancy and the main cause of their increased mortality is cardiovascular disease (CVD). The objective of this study was to determine the efficacy of resveratrol supplementation on serum glucose and CVD risk factors in individuals with SZ. Methods and Results: This is a four-week randomized, double-blind controlled trial (registration No.: NCT 02062190) in which 19 men with a diagnosis of SZ, aged 18 to 65, were assigned to either a resveratrol supplement group (200 mg/day) or a placebo group (200 mg/day). In short, we did not observe significant changes after resveratrol supplementation. In the placebo group, we found a significant increase in total cholesterol levels (*p* = 0.024) and in LDL-cholesterol (*p* = 0.002), as well as a decrease in body fat percentage (*p* = 0.038). The placebo group also showed an increase in triglycerides (9.19%) and a reduction in HDL-cholesterol (4.88%). In the resveratrol group, triglycerides decreased (7.64%). Conclusion: In summary, oral resveratrol in reasonably low dosages (200 mg daily) brought no differences to body weight, waist circumference, glucose, and total cholesterol. It was possible to note that the lipid profile in the placebo group worsened and, although no significant differences were found, we can assume that resveratrol might prevent lipid profile damage and that the intervention affected the lipoprotein metabolism at various levels.

## 1. Introduction

Schizophrenia (SZ) is one of the most debilitating psychiatric disorders worldwide. Patients with SZ are generally overweight or obese and have several metabolic disorders. Additionally, these patients have a reduced life expectancy, with cardiovascular disease (CVD) being the most common cause of mortality [1,2,3,4,5].

Hence, CVD causes considerable concern and several studies support a link between atherogenesis and inflammation. Low-grade inflammatory status in overweight individuals has been proposed as one of the mediating processes in the development of metabolic diseases such as CVD and diabetes [6]. In addition, abnormal levels of LDL-cholesterol, triglycerides and HDL-cholesterol are the most important risk factors for CVD [7].

Many food compounds have been reported to have anti-inflammatory and antioxidant properties for the human metabolism [6] and there is some evidence that resveratrol supplementation offers significant potential benefits in preventing disorders like CVD [8]. Resveratrol is a natural polyphenolic compound with cardioprotective, anticancer and anti-inflammatory properties [9]. It is involved in anti-atherogenic activities and vasculoprotection [10] and is found in over 70 species of plants, including grapevines (Vitis vinifera), mulberries and peanuts. Furthermore, a recent meta-analysis of 10 studies suggested that the consumption of this polyphenol lowers CVD risk [11]. On the other hand, numerous studies have suggested that the effects of oral resveratrol supplementation on cardiovascular risk factors are inconclusive [12].

According to this line of evidence, the objective of this study was to determine the efficacy of resveratrol supplementation on serum glucose and CVD risk factors in individuals with SZ.

## 2. Methods and Materials

This is a randomized, double-blind, placebo-controlled trial of 19 male volunteers, aged 18 to 65 years, with a diagnosis of SZ established by the Structured Clinical Interview for DSM-IV-Axis I Disorders (SCID-I). The research participants followed a 1-month resveratrol supplementation program or placebo which was covered by the Public Health Service at the Schizophrenia Program of Hospital de Clinicas de Porto Alegre (HCPA), Brazil. All participants had been on a stable dose of clozapine (an atypical antipsychotic) for at least 6 months and provided signed informed consent. Exclusion criteria were the use of other antipsychotic medications, a diagnosis of diabetes, and the use of medications to treat diabetes or dyslipidemia.

The participants received nutritional orientation and a diet prescription one month before starting the study protocol. A trained nutritionist with expertise in psychiatric disorders prescribed a low-fat diet with a daily intake of 20–25 kcal/kg/day. The nutritional orientation was designed to reduce the intake of sugar and saturated fat and to maintain a regular consumption of fruit and vegetables. The clinical evaluation included anthropometric measurements (weight, height, waist and hip circumference, body mass index—BMI, and body fat percentage), smoking status, physical activity, fasting glucose, and lipid profile (total cholesterol, LDL-cholesterol, HDL-cholesterol, triglycerides). All measurements were taken on the first and last day (day 1 and day 30) of the 1-month follow-up.

The subjects were prescribed two dietary supplements a day (200 mg of resveratrol or 200 mg of placebo). Resveratrol (trans-resveratrol, 98% purified) and placebo were obtained from a compounding pharmacy in Porto Alegre, RS, Brazil. The subjects were instructed to take the first supplement after the baseline measurements (day 1) and the last supplement at the end of 4 weeks (day 30). They were also instructed to maintain their usual diet and physical activity throughout the study and to abstain from foods containing substantial amounts of resveratrol (e.g., wine, red grapes, peanuts and berries). They were also advised not to take any other food supplements. The team used two practices to monitor the adherence to the study protocol: (a) weekly telephone calls during the study period; (b) a pill count on the last day (day 30).

A double-blind trial was performed as recorded in the protocol http://clinicaltrials.gov (registration No.: NCT 02062190). The Research Ethics Committee of HCPA approved this research study (registration No.: 110553). CONSORT supported the protocol for this trial.

### 2.1. Anthropometric Measurements

Nutritional evaluation was conducted by anthropometric data (weight, height, waist and hip circumference, body fat percentage) and food intake was estimated through a 24-h Recall Survey. Weight and height were measured with a calibrated digital scale and a wall-mounted stadiometer, respectively. Body mass index (BMI) was calculated as body weight in kilograms divided by the square of the height in meters (kg/m^2^). Body composition was measured using a bioimpedance analyzer (Omron BF 300). The conicity index (C-index) estimated abdominal adiposity using the formula: waist circumference (cm)/(0.109√(body weight (kg)/height (m))) [13].

### 2.2. Plasma Biochemistry

Venous blood samples were drawn from each subject after an overnight fast (12 h fast) at baseline and at the conclusion of the study. Five milliliters of blood were collected by venipuncture into a free-anticoagulant vacuum tube. The blood was immediately centrifuged at 3000× *g* for 5 min and serum was kept frozen at −80 °C until assayed. Cardiovascular risk was assessed by analyzing the lipid profile (total cholesterol, LDL-cholesterol, HDL-cholesterol and triglycerides) and glucose levels.

### 2.3. Psychopathology Severity Assessment

Trained psychologists with expertise in psychiatric disorders assessed psychopathology severity by applying the Brief Psychiatric Rating Scale (BPRS) [14]. BPRS evaluates current symptoms and positive and negative dimensions (negative scores refer to the sum of BPRS questions 3, 9, 13, 16 and positive scores refer to the sum of BPRS questions 8, 11, 12, 15).

### 2.4. Statistical Analysis

Statistical analysis was performed using SPSS 17.0 for Windows. The results were represented as the mean ± standard deviation (SD), or median and range, or as percentages (%), as indicated. Data were analyzed by *t*-test (symmetrical distribution) or Mann-Whitney test (asymmetrical distribution) for continuous variables, whereas the Fisher’s exact test was applied for categorical variables. In the pre and post intervention comparisons, the paired Student’s *t*-test was applied for normally distributed variables and the Wilcoxon test was applied for asymmetrical distribution. Pearson’s r correlation and Spearman’s rho were used to identify correlations among variables. *p* values < 0.05 (two-tailed values) were considered statistically significant.

## 3. Results

The characteristics of the subjects are described in Table 1. The participants initially formed an apparently homogeneous population in all the assessed parameters.

**Table 1:** Baseline clinical parameters of patients in the resveratrol and placebo groups.

	Resveratrol (*n* = 10)	Placebo (*n* = 9)	*p* Value *
**Age (years)**	46.40 ± 11.18	41.00 ± 7.87	0.245
**Education (in years)**	9.90 ± 3.95	10.22 ± 2.10	0.830
**Smoking (*n*(%))**	4 (40)	3(33)	1.000
**Number of cigarette/day**	15 (6–20)	30 (20–40)	0.114
**Age of onset of the disease (years)**	23.90 ± 5.58	26.44 ± 9.67	0.486
**Length of illness (years)**	22.50 ± 10.00	14.56 ± 7.92	0.074
**Clozapine dose (mg/day)**	485 ± 213.50	600 ± 180.27	0.225
**Practice exercise (*n*(%))**	8(80)	7(77.8)	1.000
**Time of exercise (min/week)**	135 (0–450)	60 (0–300)	0.152
***Anthropometric measurements***			
**Body weight (kg)**	80.96 ± 13.99	90.45 ± 22.30	0.277
**BMI (kg/m^2^)**	26.88 ± 3.94	28.43 ± 5.85	0.505
**Waist circumference (cm)**	101.80 ± 12.30	105.78 ± 17.18	0.566
**Waist to hip ratio**	0.98 ± 0.06	0.98 ± 0.07	0.947
**Conicity index**	1.36 ± 0.07	1.36 ± 0.07	0.922
**Body fat percentage (%)**	26.82 ± 6.23	28.51 ± 5.99	0.568
***Plasma biomarkers***			
**Serum glucose (mg/dL)**	98.90 ± 13.82	94.89 ± 12.43	0.517
**Total cholesterol (mg/dL)**	186.10 ± 26.96	185.56 ± 33.23	0.969
**LDL-cholesterol (mg/dL)**	110.26 ± 25.49	107.24 ± 26.98	0.805
**HDL-cholesterol (mg/dL)**	36.30 ± 2.946	39.56 ± 7.58	0.254
**TG (mg/dL)**	197.70 ± 66.73	194.33 ± 85.23	0.924
***24-h recall***			
**Energy 24 h intake (kcal)**	2057 ± 463	2119 ± 769	0.832
**Carbohydrate 24 h intake (%)**	51.8 ± 9.9	49.4 ± 9.1	0.596
**Protein 24 h intake (%)**	21.6 ± 6.2	19.3 ± 4.0	0.351
**Total lipids 24 h intake (%)**	26.6 ± 6.8	31.1 ± 7.3	0.186
**Cholesterol 24 h intake (mg/day)**	284 (81.3–473)	217 (97–637)	0.780
**Fiber 24 h intake (g/day)**	27.6 ± 6.4	25.2 ± 11.6	0.572
***Assessment of Symptoms***			
**BPRS total score**	10.5 (2–27)	13 (5–21)	0.720
**BPRS positive symptoms**	0.5 (0–5)	0 (0–9)	0.968
**BPRS negative symptoms**	2 (0–14)	3 (0–8)	0.968

BMI: body mass index; TG: Triglycerides; BPRS: Brief Psychiatric Rating Scale. Data are shown as the mean and standard deviation (SD), median and range or relative and absolute frequency. * Independent samples *t*-test (symmetrical distribution), Fisher’s exact test (categorical variables) or Mann-Whitney test (asymmetrical distribution). There are no significant differences between the two groups (resveratrol *vs.* placebo) in the listed parameters. A two-tailed *p* value < 0.05 was considered significant.

The differences found in the clinical and biochemical parameters assessed in the two groups at the end of the fourth week of the trial are shown in Table 2. Overall, these results show that the variables and markers examined were not significantly modified after resveratrol supplementation.

**Figure 1:**
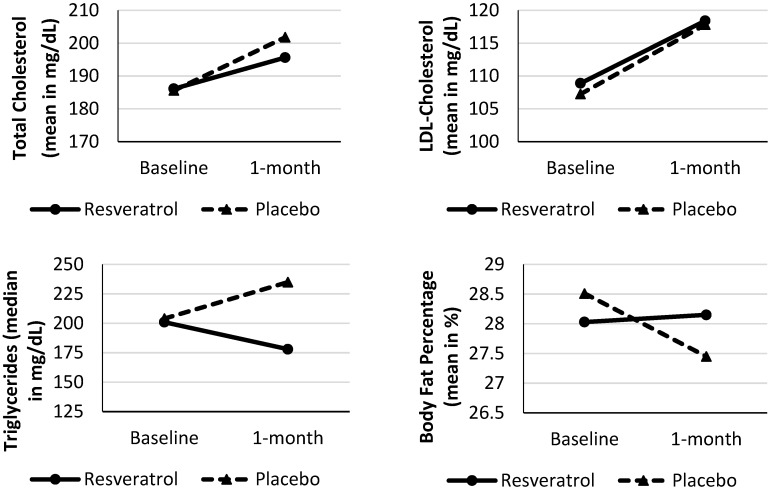
Characteristics of subjects with schizophrenia as baseline and after 1-month of resveratrol supplementation or placebo.

Total cholesterol, LDL-cholesterol, triglycerides and body fat percentage of subjects at baseline and after 4 weeks of resveratrol supplementation or placebo are shown in Figure 1. In the placebo group, we found a significant increase in the levels of total cholesterol (*p* = 0.024) and LDL-cholesterol (*p* = 0.002) as well as a decrease in body fat percentage (*p* = 0.038). The placebo group also showed a reduction in HDL-cholesterol (4.88%) and an increase in triglycerides (9.19%). The resveratrol group, on the other hand, showed a decrease in triglycerides (7.64%) (Table 3).

**Table 2:** Characteristics of subjects with schizophrenia at baseline and after 4 weeks of resveratrol supplementation or placebo.

	Resveratrol			Placebo		
Characteristics	Day 1 (Baseline)	Day 30	*p* Value *	Day 1 (Baseline)	Day 30	*p* Value *
**Exercise (min/week)**	135 (0–450)	140 (0–450)	0.463	60 (0–300)	80 (0–200)	0.461
**Weight (kg)**	80.96 ± 13.99	81.08 ± 13.93	0.845	90.45 ± 22.30	90.16 ± 23.02	0.354
**BMI (kg/m^2^)**	26.88 ± 3.94	26.83 ± 3.90	0.739	28.43 ± 5.85	28.29 ± 6.08	0.209
**Waist circumference (cm)**	101.80 ± 12.30	101.40 ± 12.03	0.583	105.78 ± 17.18	105.17 ± 17.35	0.202
**Waist to hip ratio**	0.98 ± 0.06	0.98 ± 0.07	0.581	0.98 ± 0.07	0.98 ± 0.07	0.232
**Conicity index**	1.36 ± 0.07	1.36 ± 0.07	0.454	1.36 ± 0.07	1.36 ± 0.07	0.394
**Body fat percentage (%)**	28.03 ± 5.20	28.15 ± 5.77	0.824	28.51 ± 5.99	27.45 ± 6.29	0.038 *
**Serum glucose (mg/dL)**	98.90 ± 13.82	98.80 ± 11.81	0.957	94.89 ± 12.43	92.67 ± 7.73	0.568
**Total cholesterol (mg/dL)**	186.10 ± 26.96	195.60 ± 37.64	0.149	185.56 ± 33.23	201.78 ± 40.12	0.024 *
**LDL-cholesterol (mg/dL)**	108.88 ± 26.64	118.40 ± 28.18	0.110	107.24 ± 26.98	117.80 ± 26.73	0.002 *
**HDL-cholesterol (mg/dL)**	36.30 ± 2.94	36.80 ± 3.29	0.586	39.56 ± 7.58	40.33 ± 7.00	0.718
**TG (mg/dL)**	201 (66–297)	178 (76–574)	0.508	204 (87–294)	235 (60–415)	0.236
**Energy 24 h intake (kcal)**	2057 ± 463	2095 ± 551	0.790	2119 ± 769	1842 ± 472	0.219
**Carbohydrate 24 h intake (%)**	51.8 ± 9.9	53.0 ± 6.9	0.529	49.4 ± 9.1	55.6 ± 10.3	0.255
**Protein 24 h intake (%)**	21.6 ± 6.2	20.4 ± 5.3	0.312	19.3 ± 4.0	19.0 ± 5.6	0.888
**Total lipids 24 h intake (%)**	26.6 ± 6.8	26.6 ± 5.4	0.992	31.1 ± 7.3	25.5 ± 6.1	0.154
**Cholesterol 24 h intake (mg/day)**	284 (185–446)	284 (126–434)	0.753	217 (152–377)	191 (135–298)	0.260
**Fiber 24 h intake (g/day)**	27.6 ± 6.4	27.2 ± 7.8	0.851	25.2 ± 11.6	22.2 ± 7.7	0.396
**BPRS total score**	10.5 (2.8–21.3)	16 (3–25.3)	0.475	13 (8–18.5)	14 (4–17.5)	0.917
**BPRS positive symptoms**	0.5 (0–3.3)	0.5 (0–7)	0.245	0 (0–4)	0.5 (0–2.8)	0.480
**BPRS negative symptoms**	2 (1.5–8)	3 (0–8.3)	0.933	3 (1–6)	6 (1.5–7.8)	0.197
**Non-adherence (pill returned)**	-	5 (0–12)	-	-	9 (2–18)	0.447

BMI: body mass index; TG: Triglycerides; BPRS: Brief Psychiatric Rating Scale. Data are shown as the mean and standard deviation (SD) or median and range. * Paired Student’s *t*-test was applied for normally distributed variables and Wilcoxon test for asymmetrical distribution. A two-tailed *p* value < 0.05 was considered significant.

**Table 3:** Characteristics of subjects with schizophrenia on the percentage variation of 4 weeks of resveratrol supplementation or placebo.

	Resveratrol	Placebo
**Characteristics**	Difference 1–30 (%)	Difference 1–30 (%)
**Body fat percentage (%)**	1.35 (−9.42 to 11.68)	−2.74 (−12.56 to 1.36)
**Serum glucose (mg/dL)**	0.57 (−8.27 to 6.80)	−2.12 (−20 to 17.57)
**Total cholesterol (mg/dL)**	4.55 (−13.90 to 22.22)	9.22 (−4.84 to 24)
**LDL-cholesterol (mg/dL)**	4.95 (−14.13 to 51.65)	9.14 (−2.56 to 36.19)
**HDL-cholesterol (mg/dL)**	0 (−7.14 to 13.89)	−4.88 (−19.57 to 29.27)
**TG (mg/dL)**	−7.64 (−57.78 to 103.55)	9.19 (−31.03 to 43.10)
**Energy 24 h intake (kcal)**	0 (−39.62 to 29.07)	−4.49 (−48.76 to 34.28)
**Carbohydrate 24 h intake (%)**	0 (−15.89 to 31.99)	12.11 (−25.05 to 86.04)
**Protein 24 h intake (%)**	0 (−38.7 to 13.22)	9.98 (−54.23 to 44.15)
**Total lipids 24 h intake (%)**	0 (−23.75 to 51.47)	−19.33 (−60.83 to 33.16)
**Cholesterol 24 h intake (mg/day)**	0 (−41.21 to 23.78)	−18.84 (−90.67 to 78.69)

BMI: body mass index; TG: Triglycerides. Data are shown as the median and range.

There were no changes in serum glucose, anthropometric measurements (weight, waist and hip circumference, BMI and C-index), medication dose (clozapine), number of cigarettes smoked, exercise levels, symptoms or diet intake (Table 2). Some changes in diet intake, albeit not significant, are described in Table 3.

Significant correlations are shown in Table 4. In the resveratrol group there were positive correlations between pills returned and the variation of waist circumference, C-index and body weight. There were negative correlations between pills returned and the variation of waist circumference and C-index. In addition, there were no significant side effects.

**Table 4:** Significant correlations in 4 weeks of resveratrol supplementation or placebo.

	Correlation Coefficient	
Associations	Resveratrol	Placebo
Education (years of study) *vs.* BMI	*r* = 0.747 (*p* = 0.013)	*r* = 0.361 (*p* = 0.340)
Number of cigarette/day *vs.* ∆ Body fat percentage (%)	*r*_s_ = 0.683 (*p* = 0.042)	*r_s_* = 0.164 (*p* = 0.699)
Age (years) *vs.* ∆ Body weight (kg)	*r* = −0.697 (*p* = 0.025)	*r* = −0.795 (*p* = 0.010)
Length of illness (years) *vs.* ∆ Body weight (kg)	*r* = −0.749 (*p* = 0.013)	*r* = −0.596 (*p* = 0.090)
Length of illness (years) *vs.* ∆ HDL-cholesterol (mg/dL)	*r_s_* = 0.602 (*p* = 0.066)	*r_s_* = −0.625 (*p* = 0.072)
Non-adherence (pill returned) *vs.* ∆ Waist circumference (cm)	*r_s_* = 0.816 (*p* = 0.004)	*r_s_* = −0.366 (*p* = 0.333)
Non-adherence (pill returned) *vs.* ∆ Conicity index	*r_s_* = 0.597 (*p* = 0.068)	*r_s_* = −0.412 (*p* = 0.271)
Non-adherence (pill returned) *vs.* ∆ Body weight (kg)	*r_s_* = 0.596 (*p* = 0.069)	*r_s_* = 0.055 (*p* = 0.889)
Clozapine dose (mg/day) *vs.* ∆ TG	*r* = −0.734 (*p* = 0.016)	*r* = −0.077 (*p* = 0.804)

BMI: body mass index; TG: Triglycerides. ∆: delta represents the variation of 4 weeks of resveratrol or placebo supplementation; * Pearson’s *r* correlation or Spearman’s rho were used to identify correlations among variables. *p* values < 0.05 (two-tailed values) were considered to be statistically significant.

## 4. Discussion

To our knowledge, this is the first clinical trial with resveratrol supplementation in patients with SZ. There were no differences in body weight, waist circumference, serum glucose and cholesterol in the resveratrol group.

Interestingly, we observed some changes in the lipid profile. In the placebo group, there was a significant increase in total cholesterol, LDL-cholesterol and in triglycerides, as well as a reduction in HDL-cholesterol. Meanwhile, triglycerides decreased in the resveratrol group. Thus, it is possible to note that the lipid profile worsened in the placebo group and, although no significant differences were identified, we can assume that resveratrol might prevent lipid profile damage and that the intervention affected the lipoprotein metabolism at various levels. We know that an altered lipid profile is a major risk factor for atherosclerotic cardiovascular disease [7] and that patients with SZ present significant lipid abnormalities, such as high LDL-cholesterol and triglycerides [5]. SZ treatment involves antipsychotic medications. Clozapine, in particular, is an atypical antipsychotic that is deemed safe for patients though it may produce many side effects such as cardiovascular and metabolic disorders like weight gain, dyslipidemia, diabetes, abdominal obesity, insulin resistance and metabolic syndrome [3,5]. Therefore, it is very important to control lipid damage in this population to prevent CVD mortality.

We also observed that there was a decrease in body fat percentage in the placebo group. However, this group also showed a 20% lower fat and cholesterol intake in the second assessment than they did at baseline. It is important to note that despite said reduced lipid intake in the placebo group, there was still an increase in their lipid profile markers.

Various studies in animals and *in vitro* have reported the reducing effect of resveratrol on blood pressure, serum lipids and on glucose. However, confirmation of these beneficial effects in humans through placebo-controlled clinical trials remains relatively limited and inconsistent [15,16]. Meta-analysis of available evidence was conducted with seven randomized controlled trials to obtain a conclusive result on the lipid-modulating effects of resveratrol. It was found that, regardless of the dose or duration of supplementation, resveratrol had no significant effect on any of the lipid parameters assessed: total cholesterol, LDL-cholesterol, HDL-cholesterol and triglycerides [17]. In addition, more recent meta-analysis of data from 10 randomized controlled trials suggests that resveratrol supplementation offers no benefits to CVD risk factors [12].

Qureshi *et al.* (2013) [18] studied a nutritional supplement with a combination of various naturally-occurring proteasome inhibitors (resveratrol, pterostilbene, quercetin, δ-tocotrienol, nicotinic acid). They observed that the serum levels of total cholesterol, LDL-cholesterol and triglycerides were significantly decreased in human subjects with elevated total serum cholesterol levels, but this finding did not hold true for subjects with normal total serum cholesterol levels. This demonstrates that the beneficial effects can occur in a population that already has risk factors but not in healthy individuals. On the other hand, Kjær *et al.* (2014) [19] conducted a placebo-controlled double-blind clinical trial to investigate the anti-inflammatory effects of long-term resveratrol treatment and its effects on the metabolic syndrome. In their data, resveratrol did not affect the parameters of the metabolic syndrome nor body composition or adipose tissue depots after 4 months of resveratrol treatment. Therefore, even though data from animal studies look promising, currently the number of clinical trials is too limited to make any firm statements regarding the effects of resveratrol on obesity-induced negative health outcomes in humans [15].

Our study showed that serum glucose did not change after resveratrol supplementation. In a recent meta-analysis, Liu *et al.* (2014) [20] included eleven studies comprising a total of 388 subjects and demonstrated that resveratrol significantly improves glucose control and insulin sensitivity in individuals with diabetes but does not affect glycemic measurements in individuals who do not have diabetes. The mechanisms of how resveratrol influences glucose control and insulin sensitivity in participants who do not have diabetes are unclear. One explanation is that the participants had normal baseline glucose concentrations, which fluctuate in a certain range under normal physical conditions, and resveratrol treatments might not affect the physiological regulation of plasma glucose and insulin concentrations in these subjects.

In our data, there were positive correlations between pills returned and the variation of waist circumference, C-index and body weight. This means that lack of adherence to treatment may exert an influence on results obtained, especially in weight gain and abdominal obesity. Adherence is very difficult to control in randomized clinical trials, notably in patients with psychiatric illness. Non-adherence is one of the most common causes of therapeutic failure in medicine and psychiatry. Non-adherence must therefore be considered when planning treatment strategies among psychiatric patients [21] and when promoting the use of resveratrol. Some strategies could be useful to improve adherence: a good therapeutic relationship; the patient and the family must be provided with information about the treatment; regular contact with patients under long-term treatment must be maintained [22], there should be pharmacy-driven interventions as well as educational interventions [23].

Thus, there are conflicting results as to the effect of resveratrol in humans, with some studies reporting improvements while others find no effects. There are many reasons for resveratrol’s lack of efficacy. First, in lower doses, the serum levels of resveratrol may not be adequate for a beneficial effect to be identified. Second, resveratrol has relatively low bioavailability due to its substantial and rapid hepatic metabolism. Another possible explanation is the fact that it is the total polyphenols in red wine, not resveratrol alone, that generate the beneficial effects of red wine [12]. Finally, it is difficult to establish good adherence to treatment in patients with psychiatric disorders.

Future research is needed to consider possible resveratrol benefits. Long-term clinical trials (greater than six months) with larger sample sizes are needed to confirm the impact of these and other promising studies. Despite these limitations, this study provides an additional effect of resveratrol supplementation: a tendency for the lipid profile to decrease, which in turn may help prevent risk of CVD. We believe this can improve therapeutic and clinical outcomes, and the quality of life of individuals with SZ.

## 5. Conclusions

In conclusion, we have shown that four weeks of resveratrol supplementation (200 mg/day) do not change the variables and markers examined. The lipid profile worsened in the placebo group and, although no significant differences were found, we can assume that resveratrol might prevent lipid profile damage and that the intervention affected the lipoprotein metabolism at various levels. These findings indicate that resveratrol deserves additional attention for the clinical care of SZ due to its role in comorbidity prevention and its protective effect on CVD.
